# Peripheral Inflammatory Parameters in Late-Life Depression: A Systematic Review

**DOI:** 10.3390/ijms17122022

**Published:** 2016-12-02

**Authors:** Mónica Martínez-Cengotitabengoa, Lucía Carrascón, John T. O’Brien, María-José Díaz-Gutiérrez, Cristina Bermúdez-Ampudia, Kenji Sanada, Marta Arrasate, Ana González-Pinto

**Affiliations:** 1 Biomedical Research Centre in Mental Health Network (CIBERSAM), BioAraba, Health Research Institute, Araba University Hospital, 01004 Vitoria, Spain; Lcarrascon@alumni.unav.es (L.C.); cristina.bermudezampudia@osakidetza.eus (C.B.-A.); ksanappu@gmail.com (K.S.); anamaria.gonzalez-pintoarrillaga@osakidetza.eus (A.G.-P.); 2 Psychobiology Department, National Distance Education University (UNED), 01008 Vitoria, Spain; 3 Gregorio Marañón University General Hospital, 28007 Madrid, Spain; 4 Department of Psychiatry, University of Cambridge, Cambridge CB2 0SZ, UK; john.obrien@medschl.cam.ac.uk; 5 Community Pharmacist, Getxo, 48992 Vizcaya, Spain; Marijo72@euskalnet.net; 6 Department of Psychiatry, Showa University School of Medicine, Tokyo 157-8577, Japan; 7 RSMB-CSM Uribe, 48990 Algorta-Getxo, Spain; MARTA.ARRASATEGIL@osakidetza.eus; 8 Neurosciences Department, University of the Basque Country, 48940 Vizcaya, Spain

**Keywords:** inflammation, aging, depression, late-life

## Abstract

Depressive disorders appear relatively frequently in older patients, and therefore represent an important disease burden worldwide. Given the high levels of inflammatory parameters found in depressed elderly patients, the “inflammaging” hypothesis is gaining strength. In this systematic review, we summarize current evidence regarding the relationship between inflammatory parameters and late-life depression, with a unique focus on longitudinal studies to guarantee temporality. According to the data summarized in this review, the levels of some proinflammatory parameters—especially interleukin (IL)-8, IL-6, and tumor necrosis factor (TNF)-α—could serve as biomarkers for the future development of depressive symptoms in elderly patients. Proinflammatory cytokines seem to be associated with the future development of clinically significant depression, irrespective of baseline scores, thus indicating that inflammation temporally precedes and increases depression risk. As insufficient research has been conducted in this field, further prospective studies are clearly warranted.

## 1. Introduction

Life expectancy has continued to increase worldwide for several decades, especially in developed countries, thus a proportional increase in diseases typically affecting older people is expected to be observed in the coming years [1]. Depressive disorders—both major depressive disorder and, very often, subthreshold depressive symptoms, either alone or in combination with other conditions—are among the most common psychiatric disorders in the elderly, and they often do not respond as well to treatment than those of younger ages. Despite extensive research aimed at improving our understanding of the pathophysiology of these diseases, they still represent a significant cause of disease burden worldwide. As such, more research is required, especially to elucidate the mechanisms triggering the disease, in order to develop new preventive and treatment approaches.

Three etiological hypotheses have been proposed for depressive disorders in the elderly [2]: the “degenerative” hypothesis, according to which depression probably precedes the onset of cognitive impairment and dementia [3]; the “vascular” hypothesis, due to the findings of white matter lesions with neuroimaging studies in patients with late-life depression [4,5]; and the “inflammatory” hypothesis, based on the high levels of inflammatory parameters found in depressed elderly patients [6], which has led to the term “inflammaging” [7]. According to this latter hypothesis, aging shifts the body into a proinflammatory state mediated by an increased immune response at the peripheral level, impaired immune communication between the central nervous system and periphery, and an increased and discordant central nervous system response [6]. All these changes lead to a chronic state of neuroinflammation characterized by the continuous production of proinflammatory cytokines [8]. Indeed, the administration of cytokines has been shown to lead to depressive-like symptomatology [9,10] that can be lessened by antidepressants [11], and antidepressant treatment decreases the stimulated production of proinflammatory cytokines in depressed patients [12]. Moreover, as the degenerative, vascular, and inflammatory hypotheses are not mutually exclusive [13], this overlap of models suggests that inflammation can have a functional impact on brain structure [14].

Findings concerning inflammatory parameters and clinical correlates of depression have been contradictory, this being attributed to differences in study designs, the inherent variability in measurements of biological systems, and the different confounding factors considered in the statistical analyses. While multiple studies have reported positive associations between inflammatory mediators and late-life depression, some have not [15]. Most studies assessing the relationship between inflammation and late-life depression are cross-sectional [16,17,18,19,20,21] or case-control studies [22,23], hence it is unclear whether inflammatory conditions precede or follow the onset of symptoms.

The aim of the present review is to summarize the current evidence concerning the relationship between inflammatory parameters and late-life depression by reviewing longitudinal studies of “good methodological quality”; in other words, those articles that met minimum criteria of internal and external validity, provided a good description of their objectives, had a design in accordance with these objectives, a sufficiently large sample size, and presented their results clearly.

## 2. Literature Search

Publications were identified by way of a systematic literature search conducted initially in September 2014, which was repeated in May 2015; the review is reported in accordance with the PRISMA (Preferred Reporting Items for Systematic Reviews and Meta-Analyses) statement [24]. The search was conducted for papers published between January 2000 and May 2015 using the PubMed, Embase, PsychINFO, Trip, and Web of Science databases, and the electronic database searches were supplemented by cross-referencing of selected articles and a manual search.

The search terms “inflammation”, “depression”, “aged”, “aging”, and “elderly” were used to search for publications reporting prospective studies assessing inflammatory parameters in cohorts of elderly subjects (≥60 years old), exploring their relationship with major depressive disorder and not with other conditions. Publications in English, French, or Spanish were included. Studies reporting only cross-sectional data and genetic studies were excluded. The purpose of including only longitudinal studies was to explore temporality, which provides greater consistency to a causal model.

Selected publications were assessed by two independent raters using a critical appraisal tool platform [25]. This tool allows an in-depth analysis of all the key points that determine a study’s methodological quality by evaluating different aspects of the quality of the article (internal and external validity, clear objectives, sample size, presentation of results, etc.), and thereby classifies studies as having high, medium, or low methodological quality. Only papers classified as being of high or medium quality were included in this review. The tool also helps the reader to summarize the main characteristics of a paper.

## 3. Results

**Figure 1:**
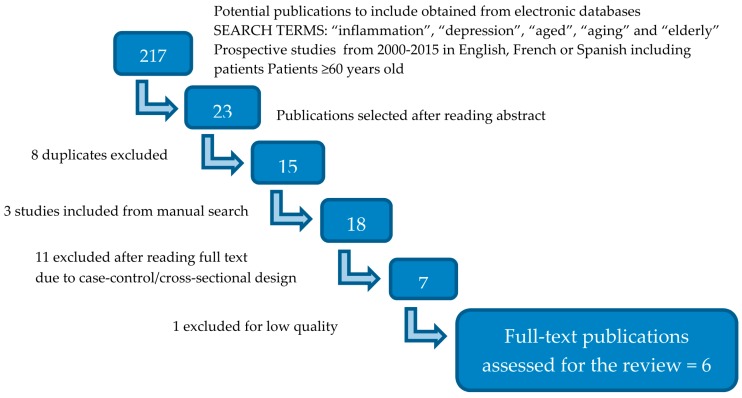
PRISMA (Preferred Reporting Items for Systematic Reviews and Meta-Analyses) flowchart of publications selection procedure.

The initial literature search identified 217 publications potentially eligible for inclusion, of which 23 were selected after analyzing each of the abstracts. After excluding 8 of these for being duplicates, and including a further 3 from the manual search, we obtained a sample of 18 papers for which we proceeded to carefully read the full text. Eleven of these papers were excluded because they had a cross-sectional or case-control design, and 1 was excluded due to its low methodological quality. Finally, we selected 6 manuscripts, which were examined in depth, for inclusion in our systematic review (Figure 1).

Only six studies met the selection criteria and had prospectively evaluated the relationship between peripheral inflammatory biomarkers and depression in older people. Samples varied in size from 263 to 1037 participants and the mean age range varied from 61 to 85 years. All but one of the studies evaluated the influence of biological measures on depressive symptoms [26,27,28,29,30], while the other study evaluated the relation between these variables in both directions [31]. Five of the studies were conducted in Europe, one in Australia, and one in the USA. The main characteristics of these studies are listed in Table 1.

The immunological markers examined in the articles reviewed are listed in Table 2.

Baune et al. [26] followed up on a cohort of 1037 Australian non-demented elderly patients for two years. Of these, 71 patients already had mild to moderate depressive symptoms at the baseline assessment, and 42 developed such symptoms during follow-up. The peripheral parameters assessed were levels of interleukin 1β (IL-1β), IL-6, IL-8, IL-10, IL-12p70, C-reactive protein (CRP), plasminogen activator inhibitor-1 (PAI-1), vascular adhesion molecule-1 (sVCAM-1), serum amyloid-A (SAA), and tumor necrosis factor-α (TNF-α). At baseline, elevated levels of IL-8 and IL-6 were related to higher levels of depressive symptoms, as assessed using the Geriatric Depression Scale (GDS) (odds ratio (OR) = 1.042; *p* = 0.025 and OR = 1.041; *p* = 0.035, respectively). Interestingly, elevated levels of IL-8 at baseline predicted significantly higher GDS scores at follow-up (OR = 1.043; *p* = 0.038) and were also associated with the first onset of mild to moderate depressive symptoms over two years (OR = 1.052; *p* = 0.038). Moreover, IL-12p70 showed a significant inverse relationship with GDS scores (OR = 0.992; *p* = 0.010).

In their population-based study, Bremmer et al. [27] included 1285 participants aged 65 and over living in the Netherlands and followed them up for one year. The authors assessed IL-6 and CRP at baseline and depressive symptoms using the Center for Epidemiological Studies—Depression (CES-D) scale. Patients with depression scores ≥ 16 were thoroughly assessed according to Diagnostic and Statistical Manual of Mental Disorders (DSM)-III criteria [35] for a diagnosis of major depressive disorder. Although no significant associations were found with CRP levels, high levels of IL-6 (above 5 pg/mL) were associated with major depression during the follow-up (OR = 2.49 (1.07–5.80)) both in first and recurrent episodes (OR = 2.87 (0.75–10.9) and OR = 2.17 (0.86–5.49), respectively). Furthermore, the authors found an OR of 3.18 for depression in patients with high IL-6 levels. With regard to symptom profile, they found no association with inflammatory parameters.

Forti et al. conducted an interesting study in Italy following up a sample of 704 elderly individuals for four years and assessed the incidence of depressive symptoms using the GDS [28]. Only TNF-α levels showed an association with incident depression, with this association having a U-shaped form. Subjects with detectable TNF levels of up to 10 pg/mL were at a lower risk of developing depressive symptoms than subjects with undetectable TNF levels (*p* = 0.015). No association was found for TNF values higher than 10 pg/mL (*p* > 0.05).

Similarly, Milaneschi et al. conducted a follow-up study of a sample of 778 elderly patients, with two follow-up assessments (three and six years after evaluating their baseline levels) of several inflammatory parameters [29]. These authors investigated the presence of depressive symptomatology using the Center for Epidemiological Studies—Depression scale at both follow-up assessments, and concluded that the IL-1 receptor antagonist (IL-1ra) predicted the development of depressed mood in the long term, this association being detectable at six years but not earlier (i.e., three-year assessment).

In contrast, and with regard to IL-1ra, van den Biggelaar et al. found that higher baseline levels of this soluble receptor were related to a lower risk of developing depression, as evaluated in a five-year follow-up assessment [30]. These authors also reported a significant relationship between IL-1β and CRP levels at baseline and increases in depressive mood, as measured using the GDS scale.

The aim of all the studies discussed thus far was to evaluate the influence of various inflammatory variables on the development of depressed mood over a given follow-up period. In contrast, the study published by Stewart et al. in 2009 followed a bidirectional approach; in other words, these authors also explored the association of baseline depressive symptoms with IL-6 and CRP levels over a six-year period [31]. They found that neither baseline IL-6 nor CRP levels behaved as predictors of the change in Beck Depression Inventory-II (BDI-II) scores during follow-up. Interestingly, the baseline Beck Depression Inventory-II scores correlated positively with changes in IL-6 levels over the follow-up period, but not with changes in CRP levels.

## 4. Discussion

According to the data summarized in this review, the levels of some proinflammatory parameters—namely IL-8, IL-6, and TNF-α—could serve as biomarkers for the future onset of depressive symptoms in elderly patients. Having said that, their prognostic value should be explored further given that, for example, Stewart et al. [31] did not replicate the previous findings of Bremmer [27] concerning IL-6. Such inconsistencies could be due to age differences between samples (61 vs. 75.4 years) and to the different tools used to assess depressive symptomatology (BDI-II vs. CES-D). The discrepancies found support the need for further studies using similar samples and designs that replicate and confirm the results of previous studies. The relevance of TNF-α is supported by the recent review of Bortolato et al., who indicate that TNF-α antagonists play an important role in mitigating depressive symptoms and improving cognitive deficits in elderly patients [36]. There is also evidence that IL-6 and TNF-α could act as mediators in the incidence of depressive symptoms in patients with some other diseases, such as coronary artery disease [37] or multiple sclerosis [38]. TNF-α may contribute to the pathophysiology of depression by activating the hypothalamic–pituitary–adrenal (HPA) axis and neuronal serotonin transporters and by stimulating the enzyme indoleamine-2,3-dioxygenase (IDO), which causes tryptophan depletion [39].

With respect to IL-8, there is some evidence that this interleukin is related to depressive symptoms both at baseline and two years later, although, to the best of our knowledge, only one trial [26] has assessed it and the ORs reported were not very conclusive (OR = 1.36 (95% CI = 1.021–1.802, *p* = 0.035) and 0.94 (95% CI = 0.67–1.32, *p* = 0.719), respectively). In line with this, a prospective study reported that the associations between the onset of depression and physical comorbidities in elderly subjects were significant in the presence of higher IL-8 production [40]. In addition, one postmortem study found that IL-8 expression was upregulated in Brodmann area 10 (prefrontal cortex) of brains from psychotropic drug-free individuals with a history of major depressive disorder compared to its expression in matched controls [41]. On the other hand, a recent systematic review concluded that the findings from cross-sectional studies regarding the relationship between depression and serum IL-8 levels in nongeriatric populations remained unclear [42], although a study conducted in 2003 found that IL-8 levels significantly and positively correlated with current depressive symptoms in a sample of 53 young men [43]. Considering these previous studies together, we conclude that further research is needed to elucidate the relationship between IL-8 and the development of depressive symptoms in elderly subjects.

With regard to IL-6, this interleukin showed a stronger association with the development of depressive symptomatology after one year (OR = 3.18), although no distinction was made between the different endophenotypes of the disease [27]. Similarly, a prospective population-based study found higher IL-6 and IL-8 levels in the cerebrospinal fluid (CSF) of elderly depressed women, this fluid being a better tissue for representing the central nervous system than peripheral blood due to its proximity to said system [44]. These results may be supported by previous meta-analyses [42,43,44].

Dowlati et al. observed significantly higher concentrations of IL-6 in depressed individuals than in healthy controls. In addition, Endrighi et al. showed an association between negative mood and stress-induced IL-6 responses in healthy young individuals [45]. IL-6 strongly activates the HPA axis and, therefore, may also contribute to the hypercortisolism observed in depression [46]. In regard to the type of depressive symptoms, Capuron et al. only observed that IL-6 appeared to be related to the “reduced appetite” score of the Montgomery–Asberg Depression Rating Scale in elderly subjects [47]. Moreover, a recently published review concluded that IL-6 is the cytokine most robustly associated with suicidal ideation, suicides, and nonfatal suicide attempts [48], thereby suggesting an important role of this interleukin in this aspect of depression. The authors found that the results for other inflammatory parameters were inconclusive.

With regard to TNF-α, the level of this protein appeared to have a U-shaped association with risk of incident depressive symptoms in elderly individuals [28]. In line with this, a prospective study found that the relationships between the onset of depression and physical disorders in elderly people were significant in the presence of higher TNF-α production, similar to production of IL-8, as cited previously [40]. Additionally, the findings from previous meta-analyses indicate that depressed individuals have significantly higher TNF-α and IL-6 concentrations than controls [49,50].

IL-1ra should be investigated more exhaustively, given the inconsistent results found in our systematic review. Thus, Milaneschi et al. [29] reported that IL-1ra predicted the development of depressed mood over a six-year follow-up period, whereas van den Biggelaar et al. [30] found that increased IL-1ra levels were related to a lower risk of developing depression. These divergent findings may be due to differences in the methodology and study samples; in particular, the disparity between direct in vivo serum measurements [29] and ex vivo whole sample blood lipopolysaccharide stimulation [30], and the gap in mean ages between the trials [51]. A systematic review concluded that IL-1ra concentrations were frequently upregulated in depressed patients [52]. In line with this review, previous studies have found higher levels of serum IL-1ra in depressed patients than in healthy populations [44,49,50,53].

Additionally, in population-based research in middle-aged individuals, higher IL-1ra levels have been associated with elevated depressive symptoms [51,54]. Similarly, a cohort study of healthy middle-aged men found evidence that higher IL-1ra levels may be associated with depression and increased mortality [55]. Furthermore, at the preclinical level, research conducted in IL-1ra knockout mice, which display an aging-induced anxiety-like phenotype, suggests an interaction between this receptor and aging in the development of depressive symptoms [56]. Taking these findings as a whole, we conclude that further research is also required to explore the association between IL-1ra and the development of depressive symptomatology in elderly people.

As mentioned above, one plausible explanation for why all these inflammatory variables influence the development of depressive symptoms is that the enzyme indoleamine-2,3-dioxygenase (IDO) is induced upon immune activation. When induced, this enzyme catalyzes the conversion of tryptophan into kynurenine, thus causing depletion of the tryptophan available to synthesize serotonin [39,57]. Furthermore, alterations in serotonergic, noradrenergic, and glutamatergic neurotransmission have also been related to low-level neuroinflammation and may be involved in the development of depressive symptoms [58].

It should be noted here that most of the articles reviewed were able to show that proinflammatory cytokines are associated with the future development of clinically significant depression, independent of baseline scores, thus indicating that inflammation temporally precedes and increases depression risk, although very few studies have addressed this subject to date.

The methodology, follow-up period, set of pro-/anti-inflammatory parameters evaluated, and tool used to assess depressive symptoms vary considerably between the studies included in this systematic review. As such, there is a clear need to conduct further research into this issue with uniform criteria across studies, including the inflammatory parameters highlighted in this review and a common clinical scale to measure depressive symptoms. There is also a need to study inflammatory markers in CSF and—as central inflammation can be assessed using positron emission tomography (PET) ligands specific for the translocator protein (TPSO)—to undertake in vivo PET studies both during depression and after recovery.

## Figures and Tables

**Table 1:** Main characteristics of cohort studies included in the review.

Study	Country	Follow-Up	Sample	Age Mean (SD)	Inflammatory Parameters Assessed *	Directionality of the Explored Relationship	Measure of Depression **	Change Score	Confounders Considered	Results	Potential Bias
Baune, 2012 [26]	Australia	2 years	1037 non-depressed and non-demented individuals	78.8 (4.8)	IL-1β, IL-6, IL-8, IL-10, IL-12p70 CRP, PAI-1, sVCAM-1, SAA, TNF-α	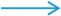	GDS [32]	Cutoff of ≥6 for depression	Gender, age, years of education, total number of medical disorders, cardiovascular disorders, endocrine disorders, smoking, BMI, current antidepressants, NSAIDs, statins, Mini Mental State Examination and Diabetes Mellitus	IL-8 and IL-6 at baseline were directly related to depression severity at baseline IL-8 at baseline directly predicted depressive symptoms at follow-up IL-12p70 at baseline showed an inverse relationship with GDS score at follow-up Remitted depression at baseline was associated with baseline PAI-1 levels	A structured interview was not used to determine the diagnosis of depression
Bremmer, 2008 [27]	The Netherlands	1 year	1285 elderly people	75.4 (6.6)	Il-6, CRP	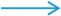	CES-D [33] and NIMH-Diagnostic Interview Schedule [34]	Cutoff of ≥16 for depression	Age, gender, educational level, household composition, current smoking, alcohol intake, medication use, several cardiovascular risk factors and chronic diseases	IL-6 plasma levels, but not CRP, were independently associated with major depression (either first or recurrent episodes). OR = 3.18 for depression in patients with high IL-6 levels No association between inflammatory parameters with respect to symptom profiles	
Forti, 2010 [28]	Italy	4 years	704 elderly people	73.4 (6.1)	IL-6, CRP, ICAM-1, ACT, TNF-α	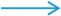	GDS, DSM-IV criteria	Cutoff of ≥10 for depression	Years of education, BMI, cardiovascular disease, comorbidity, physical disability, anti-inflammatory drug use	U-shaped association between TNF-α and risk of incident depressive symptoms	A structured interview was not used to determine the diagnosis of depression
Milaneschi, 2009 [29]	Italy	3 and 6 years	778 elderly people	75 (7)	IL-6, sIL-6r, IL-1β, IL-1ra, TNF-α, IL-18, CRP	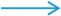	CES-D	Cutoff of ≥20 for depression	Age, gender, site, years of education, smoking habit, alcohol use, Mini Mental Stare Examination Score, BMI, number of drugs, use of NSAIDs, cardiovascular diseases and physical activity	IL-1ra predicted the development of depressed mood over 6 years’ follow-up; effect not detectable at the 3-year assessment	A structured interview was not used to determine the diagnosis of depression The study design did not allow depressive episodes started and remitted between follow-up visits to be detected
Stewart, 2009 [31]	USA	6 years	263 healthy adults (50–70 years)	61 (4.8)	IL-6, CRP	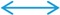	BDI-II [35]	Change in the BDI-II score	Blood pressure, BMI, HDL cholesterol, triglycerides, fasting glucose, fasting insulin, diabetes, rheumatoid arthritis, carotid intima-media thickness, smoking status, alcohol intake and physical activity	Neither baseline IL-6 nor CRP were predictors of BDI-II change during the follow-up Baseline BDI-II was positively associated with IL-6 change but was not a predictor of CRP change	Results not generalizable to racial groups other than white people, because only 35 non-white people were included in the study
Van den Biggelaar, 2007 [30]	The Netherlands	5 years	599 aged people	85 (0)	IL-1β, IL-1ra, IL-6, IL-10, CPR, TNF-α	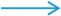	GDS	Cutoff of ≥5 for depression	Gender, education, Mini Mental State Examination Score, comorbidity, disability, current smoking, BMI, serum levels of albumin, CRP and LPS-induced cytokine	Increased levels of IL-1β and CPR were significantly related to an increase in depressive symptoms Increased levels of IL-1ra were related to a decrease in the risk of developing depression	A structured interview was not used to determine the diagnosis of depression

* IL: Interleukin; CRP: C-reactive protein; PAI-1: plasminogen activator inhibitor-1; sVCAM-1: serum vascular cell adhesion molecule-1; SAA: serum amyloid A; TNF-α: tumor necrosis factor-α; sIL-6r: soluble IL-6 receptor; IL-1ra: IL-1 receptor antagonist; ICAM-1: intracellular adhesion molecule-1; ACT: α-1-antichymotrypsin; ** GDS: Geriatric Depression Scale [32]; CES-D: Center for Epidemiological Studies-Depression Scale [33]; BDI-II: Beck Depression Inventory-II [35].

**Table 2:** Immunological markers and their role in inflammatory processes assessed in the articles reviewed.

Proinflammatory parameters	IL-1β: Interleukin 1β
	IL-6: Interleukin 6
	sIL-6r: soluble IL-6 receptor
	IL-8: Interleukin 8
	IL-12p70: IL-12 heterodimer
	IL-18: Interleukin 18
	CRP: C-reactive protein
	TNF-α: Tumor necrosis factor α
	ICAM-1: Intercellular adhesion molecule 1
	ACT: α 1-antichymotrypsin
	PAI-1: Plasminogen activator inhibitor-1
	sVCAM-1: Soluble vascular cell adhesion molecule 1
Anti-inflammatory parameters	IL-1ra: IL-1 receptor antagonist
	IL-10: Interleukin 10
